# Activated clotting time-guided heparinization during open AAA surgery: a pilot study

**DOI:** 10.1186/s40814-024-01500-9

**Published:** 2024-05-08

**Authors:** Liliane C. Roosendaal, Max Hoebink, Arno M. Wiersema, Jan D. Blankensteijn, Vincent Jongkind

**Affiliations:** 1 Department of Vascular Surgery, Dijklander Ziekenhuis, Maelsonstraat 3, 1624 NP Hoorn, The Netherlands; 2 Department of Vascular Surgery, Amsterdam UMC, Location VUmc, De Boelelaan 1117, 1081 HV Amsterdam, The Netherlands; 3 Amsterdam Cardiovascular Sciences, Microcirculation, Amsterdam, The Netherlands; 4 Amsterdam Cardiovascular Sciences, Atherosclerosis & Ischemic Syndromes, Amsterdam, The Netherlands

**Keywords:** Heparin, Activated clotting time, Aneurysm, Non-cardiac arterial procedures

## Abstract

**Background:**

Arterial thrombo-embolic complications (TEC) are still common during and after non-cardiac arterial procedures (NCAP). While unfractionated heparin has been used during NCAP for more than 70 years to prevent TEC, there is no consensus regarding the optimal dosing strategy. The aim of this pilot study was to test the effectiveness and feasibility of an activated clotting time (ACT)-guided heparinization protocol during open abdominal aortic aneurysm (AAA) surgery, in anticipation of a randomized controlled trial (RCT) investigating if ACT-guided heparinization leads to better clinical outcomes compared to a single bolus of 5000 IU of heparin.

**Methods:**

A prospective multicentre pilot study was performed. All patients undergoing elective open repair for an AAA (distal of the superior mesenteric artery) between March 2017 and January 2020 were included. Two heparin dosage protocols were compared: ACT-guided heparinization with an initial dose of 100 IU/kg versus a bolus of 5000 IU. The primary outcome was the effectiveness and feasibility of an ACT-guided heparinization protocol with an initial heparin dose of 100 IU/kg during open AAA surgery. Bleeding complications, TEC, and mortality were investigated for safety purposes.

**Results:**

A total of 50 patients were included in the current study. Eighteen patients received a single dose of 5000 IU of heparin and 32 patients received 100 IU/kg of heparin with additional doses based on the ACT. All patients who received the 100 IU/kg dosing protocol reached the target ACT of > 200 s. In the 5000 IU group, TEC occurred in three patients (17%), versus three patients (9.4%) in the 100 IU/kg group. Bleeding complications were found in six patients (33%) in the 5000 IU group and in 9 patients (28%) in the 100 IU/kg group. No mortality occurred in either group.

**Conclusions:**

This pilot study demonstrated that ACT-guided heparinization with an initial dose of 100 IU/kg appears to be feasible and leads to adequate anticoagulation levels. Further randomized studies seem feasible and warranted to determine whether ACT-guided heparinization results in better outcomes after open AAA repair**.**

## Key messages regarding feasibility

- What uncertainties existed regarding the feasibility?
  The aim of this pilot study was to investigate whether an activated clotting time (ACT)-guided heparinization strategy with an initial heparin dose of 100 IU/kg is effective and safe during open AAA surgery. The feasibility of adherence to the heparinization protocol, adherence to the monitoring protocol, and the level of perprocedural anticoagulation using the ACT were tested.
- What are the key feasibility findings?
  Results showed that the ACT-guided heparinization protocol with initial heparin doos of 100 IU/kg seems safe and can be implemented successfully, leading to adequate ACT values.
- What are the implications of the feasibility findings for the design of the main study?
  The heparinization- and monitoring protocol were found to be feasible and effective. Therefore the same protocol is used for the ACTION-1 trial (clinicaltrials.gov NCT04061798), a randomized controlled trial on ACT-guided heparinization during open AAA repair, which started in March 2020.

## Background

Throughout the years, outcomes of non-cardiac arterial procedures (NCAP) have improved [1, 2]. Yet, arterial thrombo-embolic complications (TEC) are still common pre- and post-procedural, especially during open abdominal aortic aneurysm (AAA) repair [3–11]. Unfractionated heparin has been used during NCAP for more than 70 years trying to prevent TEC [12]. However, there is no consensus regarding the optimal dosing strategy [13]. Heparin is heterogeneous in composition and thereby in pharmacokinetics. It has a non-linear dose–response curve and a non-linear elimination curve [14]. Moreover, there are differences in efficacy between different brands, and even in efficacy between batches of the same brand [15]. Therefore it is hard to predict the effect of heparin in the individual patient. In cardiac interventions, both open and endovascular, heparin is administered based on the weight of the patient, and the dose is adjusted during the procedure, using the activated clotting time (ACT) [16–19]. This is in contrast to NCAP, where a single bolus of 5000 IU of heparin without monitoring the effect of heparin is most often used [20]. Results from previous cohort studies suggest that ACT-guided heparinization may potentially lead to a lower incidence of TEC [21]. Large comparative studies are lacking, however, and published studies often have heterogeneous procedure types. Open AAA repair is a well-established procedure with a relatively standardized technique with a persistently high incidence of TEC and where anticoagulation and hemostasis are of vital importance [22–28]. It is therefore important to determine the best heparinization protocol during open AAA repair.

The aim of this study was to investigate whether an ACT-guided heparinization strategy with an initial heparin dose of 100 IU/kg is effective and safe during open AAA surgery, in view of determining if further studies are feasible and warranted to investigate whether ACT-guided heparinization may lead to better clinical outcomes compared to a single bolus of 5000 IU of heparin.

## Methods

### Study design

The MANCO study (measuring the ACT during non-cardiac arterial procedures, clinicaltrials.gov identifier: NCT03426293) is a prospective, multicentre cohort study for patients undergoing NCAP and receiving intraoperative heparin with ACT monitoring. The protocol was evaluated and approved by the Medical Ethical Committee Noord Holland. Local approval was obtained in both participating centers: Dijklander Hospital in Hoorn and Amsterdam UMC location VUmc, The Netherlands.

Patients were included in the pilot study if they underwent an open repair procedure for an AAA, originating distal of the superior mesenteric artery. Patients were included between March 2017 and January 2020. Exclusion criteria were acute interventions, previous open or endovascular intervention on the abdominal aorta, administration of heparin prior to surgery, allergy to heparin, eGFR < 30 ml/min, and medical history of coagulation disorders or heparin-induced thrombocytopenia.

### Heparin protocol and anticoagulation monitoring

Patients included between March 2017 and July 2018 received a single bolus of 5000 IU of heparin. After July 2018, the heparin administration protocol was modified to ACT-guided heparinization with an initial heparin dose of 100 IU/kg in both hospitals. Therefore, between July 2018 and January 2020, patients received an ACT-guided heparinization protocol with an initial heparin dose of 100 IU/kg. The heparin bolus was administered intravenously after anesthetic induction and before arterial cross-clamping.

For the 5000 IU group, the ACT was measured 5 min after heparin administration and every 30 min thereafter until the end of the procedure.

**Fig. 1:**
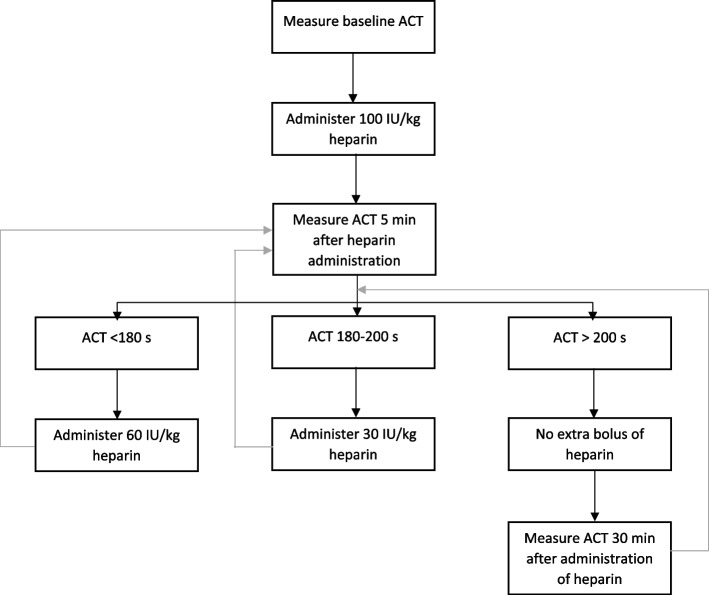
Protocol ACT-guided heparinization with an initial heparin dose of 100 IU/kg

In the 100 IU/kg group, the ACT was measured 5 min after the initial heparin gift. Depending on the ACT, additional heparin doses were administered according to the protocol (Fig. 1).

The ACT 5 min after administration of heparin in the 5000 IU group is expressed as ‘ACThep’. For the 100 IU/kg group ‘ACThep’ is defined as the first ACT > 200 s after the first heparin dosages.

Just before the end of surgery, the final ACT was measured and protamine was administered at the surgeon’s discretion. All ACT measurements were performed using the Haemostasis Management System (HMS) Plus (Medtronic®, Minneapolis, MN, USA). High-range cartridges were used during all procedures, the activator reagent was kaolin. For each measurement, a blood sample of 3 cc was drawn, obtained from an arterial line. Beforehand, 5 cc of blood was drawn and disposed of to prevent contamination of heparin residues.

### Data collection

An encrypted database, using the cloud-based Electronic Data Capture platform ‘Castor EDC’, was created [29]. The following patient characteristics were included in the database: age (years), sex, weight (kg), BMI (kg/m^2^), smoking (current, cessation, never), cardiac disease (ischemic, arrhythmia, heart failure), cardiac intervention, hypertension (systolic > 140 mmHg, diastolic > 90 mmHg), hypercholesterolemia, chronic obstructive pulmonary disease (COPD)/lung fibrosis, transient ischemic attack (TIA)/cardiovascular accident (CVA), malignancy (actual/cured), diabetes mellitus, renal function (estimated glomerular filtration rate, mL/min), peripheral arterial obstructive disease, prior abdominal surgery, prior arterial intervention. Pre-procedural anticoagulation therapy, lab results, ASA (American Society of Anesthesiologists) classification, and largest aneurysm diameter were also registered. Information about the procedure (anatomic location, total blood loss, amount of heparin given), length of stay in hospital, and all complications within the same admission or 30 days postoperative were also collected.

### Primary and secondary outcomes

The primary outcome was protocol feasibility, defined by the following outcomes: adherence to the heparinization protocol, adherence to the monitoring protocol, and level of perprocedural anticoagulation using the ACT.

The secondary outcome, for safety purposes, was the incidence of all TEC, bleeding complications, and all-cause mortality within 30 days of surgery or during the same admission in the hospital.

TEC included myocardial infarction, transient ischemic attack, cerebrovascular accident, deep venous thrombosis, pulmonary embolism, bowel ischemia, thrombo-embolic renal insufficiency (defined by RIFLE criteria: rise of serum creatinine > 100% or decrease of eGFR with 50%), athero-embolism, spinal cord ischemia, and graft thrombosis [30].

Bleeding complications were defined as European Multicentre Study on Coronary Artery Bypass Grafting (E-CABG) classification grade one or more (platelets, fresh frozen plasma, or ≥ 2 units of red blood cell transfusions) within 30 days of follow-up or during the same admission [31].

This pilot study was written according to the CONSORT (Consolidated Standards of Reporting Trials) statement, extension to pilot and feasibility trials.

### Statistical analysis

Statistical analyses were performed using the SPSS statistical software package, version 28.0 [32]. The normality of continuous variables was tested using Kolmogorov–Smirnov test. The independent *t*-test was used for continuous variables with respect to two subgroups. In the case of skewed data, the Mann–Whitney test was performed. Outcomes were expressed as mean ± standard deviation. The chi-square test was used in the case of categorical variables. Outcomes were expressed as counts and percentages. A *p* value < 0.05 was considered significant.

## Results

**Fig. 2:**
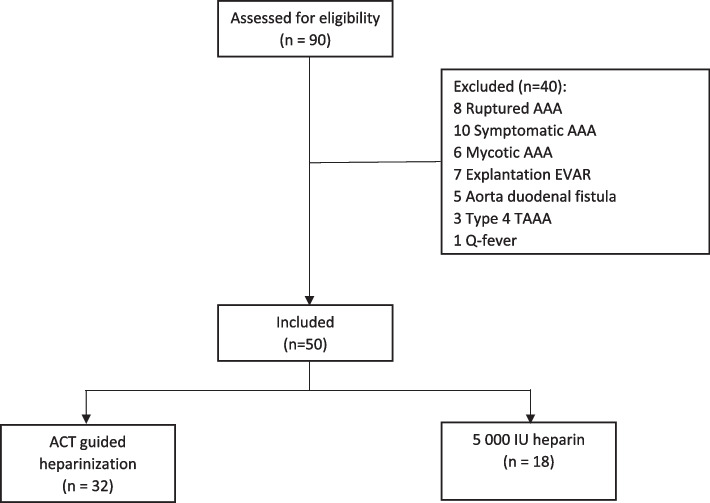
Flow diagram inclusion

A total of 50 consecutive patients were included. In the same period, 40 other patients were treated but were not included in the study (Fig. 2).

Of the included patients, 18 patients received a single dose of 5000 IU heparin, and 32 patients received ACT-guided heparinization with an initial heparin dose of 100 IU/kg.

The patient demographics are depicted in Table 1. In the 5000 IU group, the mean age in the 5000 IU group was 68, and in the 100 IU/kg group 69. The majority of patients were male (61% in the 5000 IU group and 91% in the 100 IU/kg group, *p* = *0.0*12). Chronic obstructive pulmonary disease in medical history was found more often in the 5000 IU group than in the 100 IU/kg group (50% vs 19%, *p* = 0.021). Furthermore, no significant differences in medical history were found.

**Table 1:** Patient demographics

	Heparin protocol		
	5000 IU	100 IU/kg group	
Patient demographics	(*n* = 18)	(*n* = 32)	*p* value
Age (years)	69 ± 12	68 ± 7.2	.56
Sex (male)	11 (61)	29 (91)	**.012**
Weight (kg)	76 ± 22	86 ± 19	.10
BMI (kg/m^2^)	25 ± 5.9	26 ± 4.9	.53
Medical history			
Hypertension	14 (78)	22 (69)	.50
Hypercholesterolemia	6 (33)	13 (41)	.61
Impaired renal function	1 (5.6)	1 (3.1)	.67
Diabetes	3 (17)	6 (19)	.85
Cardiac	5 (28)	13 (41)	.36
TIA/CVA	2 (11)	5 (16)	.66
COPD	9 (50)	6 (19)	**.021**
Malignancy	4 (22)	4 (130	.37
PAOD	2 (11)	9 (28)	.16
Prior arterial intervention	2 (11)	5 (16)	.66

*IU* = international unit, *n* = number of patients, *kg* = kilogram, *BMI* = body mass index, *renal function impaired* = estimated glomerular filtration rate 30 <  ×  < 40 ml/min, *cardiac* = acute infarction/ischemic, heart failure, cardiomyopathy, cardiomegaly or atrial fibrillation, *TIA* = transient ischemic attack, *CVA* = cerebrovascular accident, *COPD* = chronic obstructive pulmonary disease; *PAOD* = peripheral arterial obstructive disease

Continuous data are shown as the mean with standard deviation

Categorical variables are shown as count and percentage (%)

Bold values indicate a *p* value < .05

The preprocedural antithrombotic therapy is shown in Table 2. No differences between the groups were seen for any of the antithrombotic therapies.

**Table 2:** Preprocedural anticoagulants

	Heparin protocol	
	5000 IU	100 IU/kg
Preprocedural anticoagulants	(*n* = 18)	(*n* = 32)
None	0 (0)	3 (9.4)
Acetylsalicylic acid	12 (67)	19 (59)
Clopidogrel	3 (17)	7 (22)
Vitamin K antagonist	2 (11)	2 (6.3)
DOAC	1 (5.6)	1 (3.1)

*Note*. *n* = number of patients, *DOAC* = direct oral anticoagulant

Variables are shown as count and percentage (%)

### Adherence to protocol

All patients in the intervention group received heparin doses according to the study protocol. ACT measurements were performed at the indicated time points.

#### ACT

Mean ACT values at ‘ACThep’ are presented in Table 3. Mean ACT value in the 5000 IU group was 181 ± 40 s and in the 100 IU/kg group 230 ± 23 s (*p* = 0.003). Peak ACTs were 186 ± 37 s in 5000 IU group and 238 ± 29 s in the ACT group (*p* =  < 0.001).

**Table 3:** Mean and peak ACT per heparin protocol

	Heparin protocol		
	5000 IU	100 IU/kg	
	(*n* = 18)	(*n* = 32)	*p* value
Mean ‘ACThep’	181 ± 40	230 ± 23	**.003**
Peak ACT	186 ± 37	238 ± 29	** < .001**

*N* = number of patients, *ACT* = activated clotting time, *ACThep* = ACT 5 min after administration of heparin in the 5000 IU group. For the ACT-guided heparinization group, ‘ACThep’ is defined as the first ACT > 200 s after the first heparin dosages

Variables are shown as mean with standard deviation

Bold values indicate a *p* value < .05

### Complications

In the 5000 IU group, TEC occurred in 17% of patients (*n* = 3). One patient developed sigmoidal ischemia requiring surgical resection. The other two patients had a peripheral thrombo-embolism and underwent thrombo-embolectomy. In the 100 IU/kg group TEC occurred in 9.4% of the patients (*n* = 3). The first patient had a transient ischemic attack that was treated conservatively. The second patient had graft thrombosis and underwent a surgical revision for the right distal anastomosis. The third patient had a peripheral thrombo-embolism requiring thrombo-embolectomy. Bleeding complications were found in 6 patients (33%) in the 5000 IU group and in 9 patients (28%) in the 100 IU/kg group. No mortality occurred in both groups. Outcomes can be found in Table 4.

**Table 4:** Incidence of complications during 30-day follow-up or during the same admission

	Heparin protocol	
	5 000 IU	100 IU/kg
Complications	(*n* = 18)	(*n* = 32)
TEC	3 (17)	3 (9.4)
Bowel ischemia	1 (5.6)	0 (0)
Graft Thrombosis	0 (0)	1 (3.1)
TIA	0 (0)	1 (3.1)
Thrombo-embolism	2 (11.1)	1 (3.1)
Bleeding complications (total)	6 (33)	9 (28)
Grade 1	3 (17)	8 (25)
Grade 2	3 (17)	1 (3.1)
Cardiac complications	1 (5.6)	1 (3.1)
Renal insufficiency	1 (5.6)	4 (13)
Non-infectious fluid collection	0 (0)	4 (13)
Other minor complications	5 (28)	9 (28)
Mortality	0	0

*n* = number of patients, *TEC* = thrombo-embolic complication, *TIA* = transient ischemic attack, *E-CABG grade 1* transfusion of platelets, or transfusion of fresh frozen plasma or Octoplas, or transfusion of 2–4 red blood cell transfusions, *E-CABG grade 2* transfusion of 5–10 red blood cell transfusions or reoperation for bleeding

Variables are shown as count and percentage (%)

## Discussion

This pilot study showed that an ACT-guided heparinization protocol with an initial dose of 100 IU/kg is feasible and leads to adequate levels of anticoagulation.

To date, only two studies have been performed on the effect of heparinization during elective open AAA procedures [33, 34]. The results of both studies showed no significant differences in blood loss or thrombosis, but the results of one study demonstrated a significantly higher percentage of patients suffering a fatal perioperative myocardial infarction in the non-heparinized group.

Although no hard scientific evidence is available for the benefit of using heparin during elective AAA surgery, heparin is widely advised and used. In the current clinical practice guidelines on open AAA repair, an initial heparin dose of 50–100 IU/kg is advised [35, 36]. The ESVS guideline mentioned that the ACT can be used to measure the effect of heparin; however, no target ACT was provided or which device to use [36].

Although the optimal target ACT has not been set in guidelines, research suggests an ACT between 200 and 250 s may be preferable during NCAP, to minimize the TEC risk without compromising procedural success or increasing bleeding complications [37, 38]. Previous studies on heparinization during NCAP found a lower incidence of TEC in patients receiving an ACT-guided heparinization protocol (9%) compared to a single heparin bolus of 5 000 IU (4.3%) [4, 21]. In addition, the ACT-guided heparinization protocol, similar to the 100 IU/kg protocol used in the current study, proved to be feasible and safe during NCAP, with more patients reaching an ACT > of 200 s [39]. The results of the current study seem consistent with this literature since a lower mean ACT of 181 s was found after a bolus of 5 000 IU of heparin with TEC in 17% of patients, whereas in the 100 IU/kg a higher mean ACT of 230 s was found with TEC in 9.4% of patients.

In the Dutch Surgical Aneurysm Audit (DSAA), a Dutch registry which is mandatory for all Dutch vascular surgeons who treat patients with an AAA, the rate of serious complications was 29% for all patients who underwent elective open AAA repair from 2014 to 2016 [40]. In addition, according to the Society for Vascular Surgery AAA 2018 guidelines, the incidence of perioperative TEC was between 15 and 36% [35]. The complication rate of the patients who received the standard heparin dose of 5000 IU in this pilot study is comparable to that.

Although ACT-guided heparinization is already being used in several hospitals during NCAP, this pilot study was of importance to explore whether all patients in the intervention group actually reached the target ACT. In addition, the incidence of bleeding complications in the intervention group was investigated to assess the safety of this protocol. Results showed that the ACT-guided heparinization protocol with an initial heparin dose of 100 IU/kg seems safe and can be implemented successfully, leading to adequate ACT values.

This is a pilot study that included 50 patients from two hospitals in the Netherlands, to test for feasibility. Due to the study being underpowered and the low incidence of complications (< 5 complications per group), it was not justified to present the *p* values. In addition, the groups were not matched for patient characteristics and other risk factors. Furthermore, the present pilot study did not include any blinding strategy. Bias may very well be present.

Therefore a randomized controlled trial should be performed to determine whether ACT-guided heparinization results in safe and optimal anticoagulation during open AAA repair, with a decrease of TEC and without an increase in bleeding complications compared to a standardized bolus of 5000 IU. Derived from the amount of TEC in this pilot study (100 IU/kg:9.4%, 5000 IU: 17%), using a continuity corrected chi-square test with a two-sided alpha of 5%, 310 patients are needed in each group of the trial to achieve a power of 80%. Taking dropout into account, a total of 750 patients is needed.

The decrease in TEC might lead to less mortality and morbidity, a lower number of re-operations, or better patency, all substantially improving the patient’s quality of health, the efficiency of medical care, and the quality of vascular medical care. Based on the feasibility of this pilot study, the ACTION-1 trial (clinicaltrials.gov NCT04061798), a randomized controlled trial on ACT-guided heparinization during open AAA repair, was started in March 2020 [41].

## Conclusion

This pilot study demonstrates that ACT-guided heparinization with an initial heparin dose of 100 IU/kg appears to be feasible and leads to adequate anticoagulation levels. However, the potential beneficial effects of perioperative ACT-guided heparinization over a standardized bolus of 5000 IU on clinical outcomes (TEC and mortality) and adverse events during and after open AAA repair should be demonstrated in future large randomized studies. The ACTION-1 trial will address this [41].

## Data Availability

The data supporting the findings of this study are available from the corresponding author on request.
